# Artesunate-Loaded and Near-Infrared Dye-Conjugated Albumin Nanoparticles as High-Efficiency Tumor-Targeted Photo-Chemo Theranostic Agent

**DOI:** 10.1186/s11671-018-2700-5

**Published:** 2018-10-11

**Authors:** Hainan Yang, Zaijia Liu, Xufeng Li, Zhenfeng Zhang, Deji Chen, Hui Lian

**Affiliations:** 1 Department of Radiology, The Second Affiliated Hospital of Guangzhou Medical University, Guangzhou, 510260 China; 2 Department of Medical Image, Ezhou Central Hospital, Ezhou, 436000 China

**Keywords:** Theranostic agent, Artesunate, Fluorescence imaging, Reactive oxygen species, Indocyanine green

## Abstract

Herein, a tumor-targeted multifunctional theranostic agent was synthetized using a facile method, combining four clinically approved materials: artesunate (Arte), human serum albumin (HSA), folic acid (FA), and indocyanine green (ICG). The obtained nanocomposites (FA-IHA NPs) showed an excellent photo- and physiological stability. The ICG in the FA-IHA NPs was used not only for near infrared (NIR) fluorescence imaging, but also for photothermal and photodynamic (PTT-PDT) therapy under a single NIR irradiation. In addition, the NIR irradiation (808 nm, 1 W/cm^2^) could trigger Arte release that showed enhanced chemotherapeutic effect. Through fluorescence imaging, the cell uptake and tumor accumulation of FA-IHA NPs were observed in vitro and in vivo, analyzed by confocal microscopy and NIR fluorescence imaging in tumor xenograft mice. Based on the diagnostic results, FA-IHA NPs at 24 h post injection and combined with NIR irradiation (808 nm, 1 W/cm^2^) could efficiently suppress tumor growth through a photo-chemo combination therapy, with no tumor recurrence in vitro and in vivo. The obtained results suggested that FA-IHA NPs are promising photo-chemo theranostic agents for future clinical translation.

**Electronic supplementary material:**

The online version of this article (10.1186/s11671-018-2700-5) contains supplementary material, which is available to authorized users.

## Background

During these recent decades, imaging-guided photo-chemo therapy (IGPC) attracted great interest by many researchers, since it is a promising strategy to realize a personalized tumor therapy [1, 2]. IGPC allows the exact location of the tumor and traces the drug in vivo, guaranteeing an effective therapy and reducing side effects [3, 4]. To be effective, IGPC should have the following characteristics: (i) a multifunctional theranostic agent with both imaging and therapeutic functions is needed; (ii) the theranostic agent should be biocompatible, stable, and specific against the tumor [5–8]. The imaging diagnosis modality in the IGPC usually includes magnetic resonance imaging, photoacoustic imaging, and fluorescence imaging [9–14]. Due to the high sensitivity, favorable temporal resolution, and high signal to background ratio, fluorescence imaging was usually applied for basic research and in clinical practice [15, 16].

The photo-chemo therapy methods mainly include photothermal therapy (PTT), photodynamic therapy (PDT), and chemotherapy. Since the near infrared (NIR) irradiation is the same, PTT and PDT function can be integrated into one, making possible a selective and efficient destruction of the tumor through a laser beam. However, it was reported that photothermal and photodynamic (PTT-PDT) therapy has often the limitation of an incomplete tumor suppression, which can potentially generate a tumor relapse [17–19]. Chemotherapy, an extensively used treatment method against cancer, can effectively kill tumor cells through systemic administration, although the toxicity to nearby normal cells due to its non-specificity limits its application [20–22]. Therefore, the IGPC combination could be a great strategy to overcome the above limitations.

With the development of the nanomedicine, IGPC theranostic agents have been developed, including indocyanine green (ICG), metal-based nanoparticles, carbon nanomaterials, and polymer nanomaterials [23–27]. Among them, ICG was approved by FDA, and its use in clinical practice is reported to detect cardiac output, liver function, blood flow, and ophthalmic angiography [28, 29]. In addition, ICG has high-absorption efficiency at NIR region, thus inducing high PTT-PDT effect under a single NIR irradiation [30]. However, the following drawbacks such as instability in aqueous solution, rapid clearance in the body, the tendency to self-bleaching, and lack of targeting, severely hinder its extensive application [31, 32]. To overcome these limitations, free ICG molecules are usually carried by vehicles including micelles, polymer nanoparticles, and self-assembled protein nanostructures, to form nanocomposites [33, 34]. Although related works are available, more biocompatible and novel ICG based nanocomposites are still demanded for in vivo imaging and phototherapy.

In this work, we reported a targeted IGPC agent that covalently conjugated folic acid (FA) and ICG with human serum albumin (HSA) nanoparticles which also encapsulated the anticancer drug artesunate (Arte) (FA-IHA NPs). FA has been reported to link nanoparticles to increase their cell uptake efficiency via receptor-mediated endocytosis [17]. HSA is an endogenous protein. Because of its good biocompatibility, non-toxic, and non-immunogenicity, HSA has become one of the most exciting carriers to deliver insoluble anticancer drugs [12, 17, 31]. Arte, a natural drug extracted from *Artemisia annua*, has been proved a significant efficacy in the treatment of various cancers, such as liver cancer, lung cancer, and breast cancer [35]. The prepared FA-IHA NPs consisted of these four clinical approved materials and showed great biocompatibility and stability. As a multifunctional theranostic nanocomposite, ICG was applied as a NIR fluorescence imaging agent and phototherapy agent due to its PTT-PDT properties. Arte was highly loaded into the NPs and released by NIR irradiation for chemotherapy. Guided by the NIR imaging results, the high effect of the targeted IGPC combination was demonstrated both in vitro and in vivo. According to our results, we believe that FA-IHA NPs might be a potential versatile theranostic agent in controlled drug delivery and imaging-guided tumor-targeted combinational photo-chemo therapy.

## Methods

### Materials

N-(3-dimethylaminopropyl)-N′-ethylcarbodiimidehydrochloride (EDC), N-hydroxysuccinimide (NHS), and artesunate (Arte, ≥ 99%) were obtained from Sigma-Aldrich (USA). 4′, 6′-diamidino-2-phenylindole (DAPI) and Cell Counting Kit-8 (CCK-8) were purchased from Aladdin (Shanghai, China). NH_2_–PEG_2000_–COOH and NH_2_–PEG_2000_-FA were bought from Xi’an Ruixi Biological Technology Co., Ltd. (Xi’an, China). DMEM media and phosphate buffer saline (PBS) were provided from Gibco BRL (NY, USA). Sulfo-NHS derivative of ICG (ICG-NHS) was bought from Dojindo Laboratories (Kumamoto, Japan).

### Synthesis and Characterization of FA-IHA NPs

Artesunate was dissolved in DMSO and then added into 15 mL water. 10 mg HSA powder was added into the above solution and stirred slightly for 3 h at room temperature. After stirring, the mixture was processed by cross-linking with 150 μL 0.5% glutaraldehyde. In order to remove the redundant chemical reagents, the mixture was dialyzed against distilled water (MW cut off = 8,000–12,000 Da) for 1 day, resulting in a Arte-loaded HSA nanocomposites (Arte-HSA).

To activate the carboxylic groups of HSA, the chemical reagents EDC and NHS were added into the Arte-HSA solution. After that, the mixture was reacted with NH_2_–PEG_2000_-FA for 3 h at 4°С. Next, the ICG-NHS was added into the mixture under slight stirring for 30 min at room temperature. The purified FA and ICG-conjugated HSA nanoparticles (FA-ICG-HSA@Arte, FA-IHA NPs) were obtained by dialysis in the deionized water for 24 h. The amount of loaded Arte and ICG was detected by a UV-vis spectrophotometer. The loading efficiency = W1 / W2 × 100%, where W1 represents the weight of Arte or ICG in FA-IHA NPs, and W2 is the weight of Arte or ICG added.

The transmission electron microscopy (Hitachi, Tokyo, Japan) was used to detect the morphology of the samples. A Zetasizer (Zetasizer 3000; Malvern Instruments, Worcestershire, UK) was used to measure the size and zeta potential of the samples. A UV-vis spectrophotometer (UV-1601PC, Shimadzu, Kyoto, Japan) was applied to measure the absorbance spectra. The 808 nm single wavelength continuous wave laser (Beijing Laserwave Optoelectronics Technology Co. Ltd) was applied to conduct photothermal experiments, and the temperature was detected by a thermocouple thermometer (Fluke, USA).

### Thermal and pH-Triggered Arte Release

To determine the thermal and pH-triggered Arte release, FA-IHA NPs (50 μg/mL) was divided into three groups: (a) pH 6.5, (b) pH 7.4, and (c) pH 6.5 with NIR irradiation (808 nm, 1 W/cm^2^, 1 min pulse) at selected time points during 36 h. The released amount of Arte was determined according to the UV-vis absorption of Arte at 287 nm in the supernatant.

### Detection of Singlet Oxygen Production

1, 3-diphenylisobenzofuran (DPBF) was used to detect the singlet oxygen. 15 μL DPBF acetonitrile solution was added into pristine ICG or FA-IHA NPs solution (1.0 mL, 10 μg/mL) and mixed thoroughly, followed by 5 min of irradiation (808 nm, 1.0 W/cm^2^). The UV-vis absorption spectra were recorded at different time points, and the decrease rate of absorption at 410 nm is proportional to the singlet oxygen production.

### Cell Culture and Cellular Uptake

HepG2 cells were purchased from American Type Culture Collection and in 25 cm^2^ cell-culture flask respectively, with DMEM culture medium by adding 1% penicillin-streptomycin and 10% fetal bovine serum (FBS). HepG2 cells were kept at 37 °C in a 5% CO_2_ atmosphere.

To observe cellular uptake, HepG2 cells were cultured with free ICG, IHA NPs, and FA-IHA NPs (with 0.05 mg/mL of ICG) for 6 h. After that, PBS was used to wash the treated cells for three times. The cells were then fixed with 200 μL glutaraldehyde and stained with DAPI for 10 min. The fluorescence signals of the nanoparticles in cells were detected by using a confocal laser scanning microscope (FV300, Olympus, Japan).

To further evaluate cellular uptake, a flow cytometer (FCM, BD, Franklin Lakes, NJ, USA) was applied. As described above, the free ICG-, IHA NPs-, and FA-IHA NPs-treated cells were washed three times with PBS and digested by trypsin-EDTA. The suspended cells were directly introduced to FCM to analyze the cellular uptake ratio.

### Generation of Intracellular ROS

HepG2 cells were cultured in 12-well plates with a density of 2 × 10^5^ cells per milliliter and incubated for 24 h, followed by the addition of 1 mL of various samples including (1) PBS, (2) Arte, (3) FA-HA-NPs, (4) free ICG, (5) IHA-NPs, and (6) FA-IHA NPs solution. After a further incubation for 12 h, the cells were irradiated for 5 min (808 nm, 1.0 W/cm^2^), followed by a treatment with DCFH-DA (5 μg/mL) for another 30 min. Finally, the cells were washed thoroughly with PBS, and the production of intracellular ROS was detected quantitatively by using a cytometer and qualitatively with a Leica-inversed fluorescence microscopy.

### In Vitro Tumor Combinational Photo-Chemo Therapy

HepG2 cells were seeded in 96-well plates (2 × 10^4^ cells per well) for 24-h incubation. Free ICG, Arte, IHA NPs, and FA-IHA NPs (with 0, 5, 10, 20, and 30 μg/mL of Arte) were added into the cells. After a 6-h incubation, the old media were discarded. The treated cells were irradiated with or without an 808 nm laser (1.0 W/cm^2^, 5 min) and cultured for the next 24 h. Cell viability was measured by a classic CCK-8 assay according to the protocol.

In order to further confirm the live and dead cells after NIR treatment, the treated cells were co-stained by calcein-AM/PI. HepG2 cells were pre-seeded in 35 mm plates at a density of 1 × 10^6^ cells per plate and were treated with PBS, PBS + NIR, FA-IHA NPs, or FA-IHA NPs + NIR. After 6 h of incubation, cells were irradiated for 5 min by 808 nm laser (1 W/cm^2^) and cultured for the next 24 h. Cells were stained with calcein-AM/PI for 30 min, washed with PBS to remove excess dye solution, and then imaged using a confocal laser scanning microscope (calcein-AM lex = 488 nm, lem = 515 nm; PI lex = 535 nm, lem = 617 nm).

### Animal Model and In Vivo Fluorescence Imaging

Balb/c nude mice were obtained from The Center of Laboratory Animal Science of Guangdong Province and used under protocols approved by Guangzhou Medical University. In order to establish HepG2 subcutaneous tumors, 1 × 10^6^ HepG2 cells (in 100 μL PBS) were injected into the back of Balb/c nude mouse.

Tumor bearing mice (*n* = 5) were imaged by a commercially available IVIS Spectrum system (Caliper LifeSciences, USA) before and at 10 min, 6 h, 12 h, 24 h, and 48 h post-intravenous injection of free ICG, IHA NPs, and FA-IHA NPs.

### In Vivo Tumor Combinational Photo-Chemo therapy

Tumor bearing mice were randomly divided into different groups (*n* = 5) and were treated by PBS, Arte, FA-IHA NPs, ICG + NIR, IHA NPs + NIR, and FA-IHA NPs + NIR (with equal free Arte dose), respectively. Five-minute NIR laser (808 nm, 1 W/cm^2^) was used to irradiate the tumor region at 24 h (day 0) and 48 h (day 1) post-intravenous injection of these samples. The thermal images and temperature of the irradiated mice were recorded. During the treatment, tumor size was recorded every 4 days and calculated according to the equation: volume = (tumor length) × (tumor width)^2^ / 2. The results were shown by the relative tumor volume which was the tumor volume divided by the initial tumor volume. After treatment, major organs including heart, liver, spleen, lungs, and kidney of those mice in PBS and FA-IHA NPs + NIR groups were harvested, fixed in 4% formalin, embedded into paraffin, stained with H&E, and recorded by a digital microscope.

## Results and Discussion

### Synthesis and Characterization of FA-IHA NPs

**Fig. 1:**
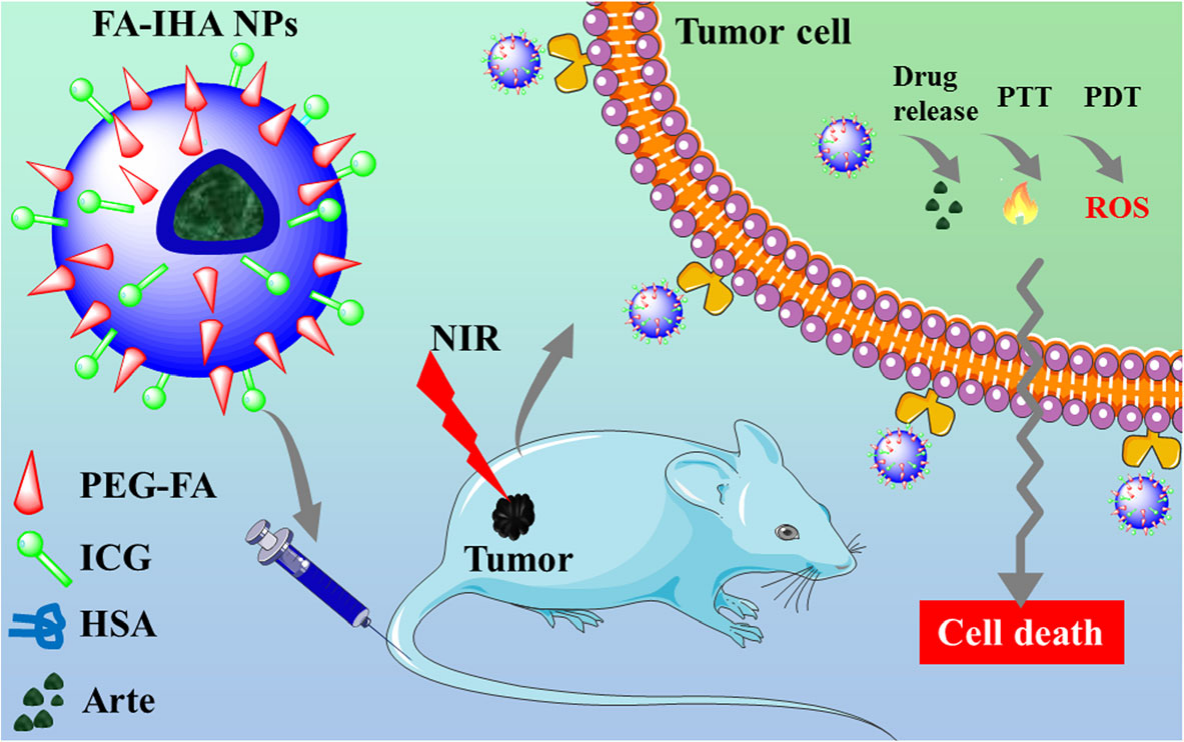
Schematic representation of FA-IHA NPs use for imaging-guided tumor-targeted combinational photo-chemo therapy in vitro and in vivo

Figure 1 illustrates the schematic use of FA-IHA NPs and their application for imaging-guided tumor-targeted combinational photo-chemo therapy. The multifunctional theranostic agents FA-IHA NPs were prepared through a simple and biocompatible self-assembly method. The conjugated ICG was employed as a NIR fluorescence imaging agent and phototherapy agent for its PTT-PDT properties. In addition, the loaded Arte exerted the chemotherapeutic effect.

**Fig. 2:**
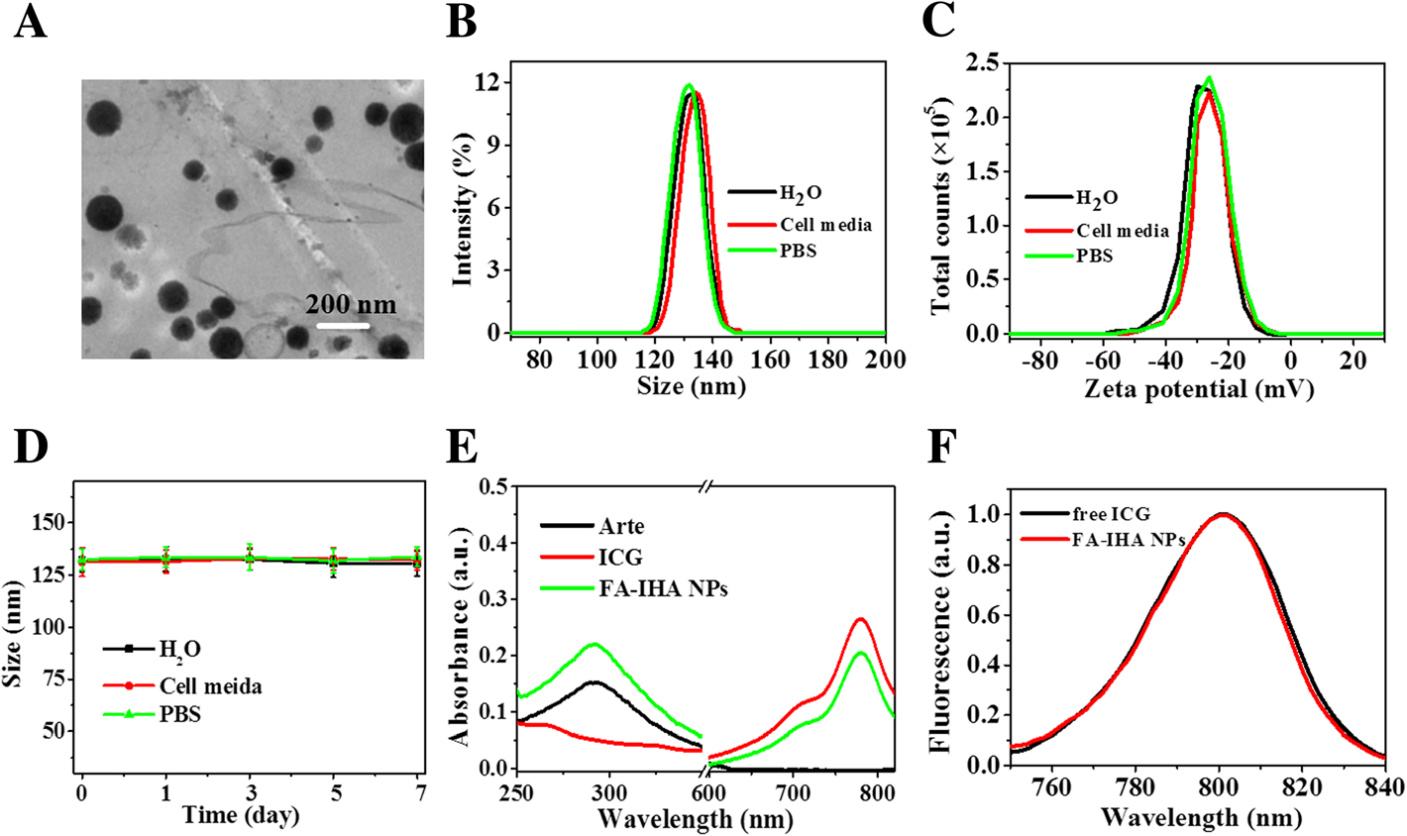
**a** TEM image of FA-IHA NPs. **b**, **c** Size and zeta potential distribution of FA-IHA NPs in water, cell medium, and PBS. **d** FA-IHA NPs size change in water, cell medium, and PBS. **e** Absorbance spectra of free ICG, Arte, and FA-IHA NPs. **f** Fluorescence spectra of free ICG and FA-IHA NPs

The TEM image of FA-IHA NPs shows a monodispersed spherical structure with a diameter of approximately 131.2 nm (Fig. 2a). This hydrodynamic diameter was confirmed as 131 ± 2.3 nm long in water, phosphate buffer saline (PBS), and cell medium (Fig. 2b), according to DLS analysis. The zeta potential of 131.2 ± 2.12 was also detected as − 29.2 ± 1.13 mV in these three media (Fig. 2c). Moreover, FA-IHA NPs diameter had no significant change over 7 days in these three media (Fig. 2d). These results indicated that the prepared FA-IHA NPs had a good stability, likely due to the PEG and HSA coating. The UV-vis-NIR spectrum of FA-IHA NPs displayed the absorption peak of both Arte and ICG (Fig. 2e), demonstrating the existence of Arte and ICG in FA-IHA NPs. Arte loading ratio was 98.6 ± 3.1%, and ICG loading ratio was 56.9 ± 2.4%. Figure 2f shows that the FA-IHA NPs had a similar fluorescence property compared to free ICG.

**Fig. 3:**
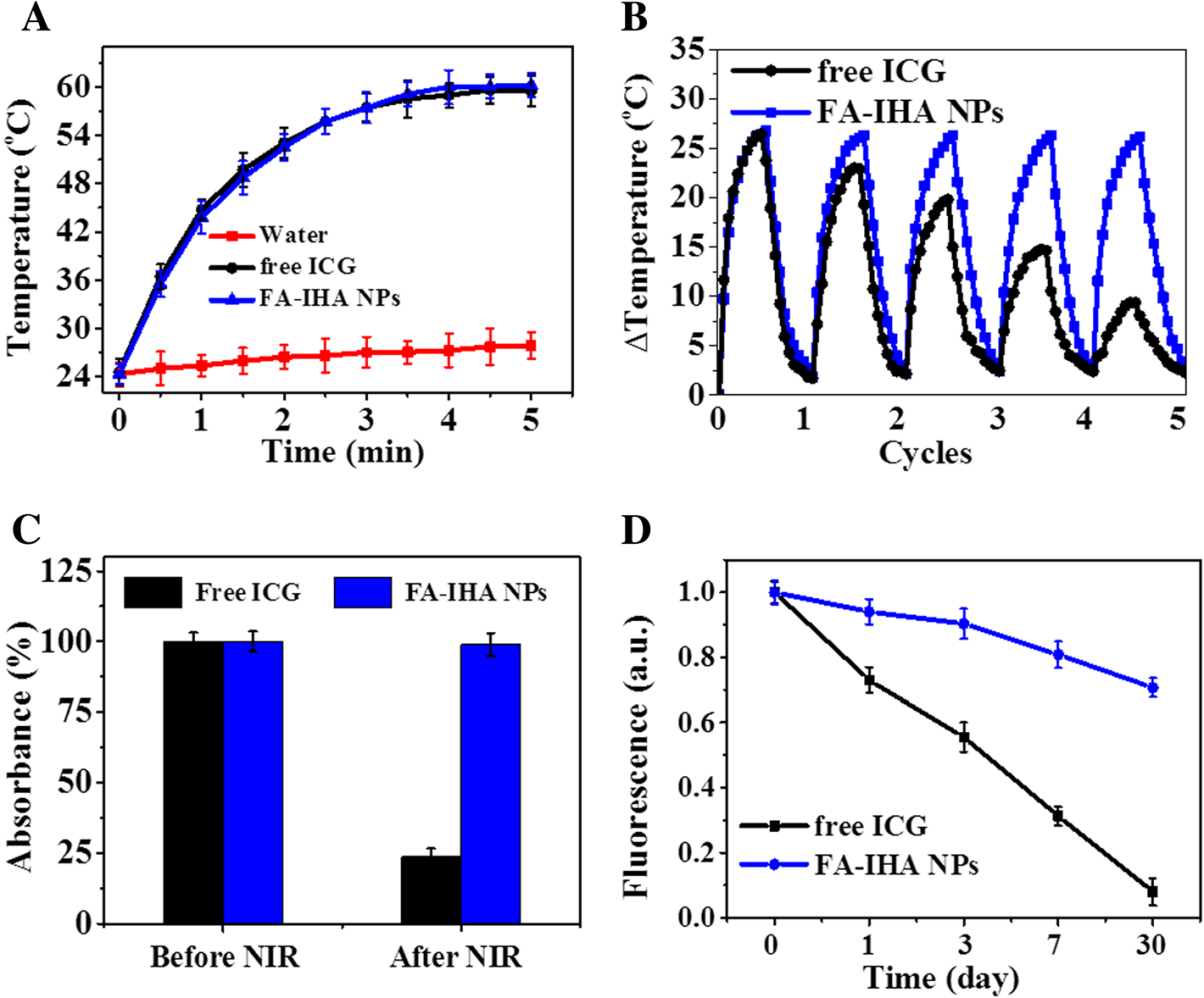
**a** Photothermal heating curves of water, ICG, and FA-IHA NPs under 5 min 808 nm laser irradiation (1 W/cm^2^). **b** Temperature variations of ICG and FA-IHA NPs after continuous 5 min of irradiation with an 808 nm laser for 5 cycles. **c** The absorption change of FA-IHA NPs at 780 nm before and after irradiation with an 808 nm NIR laser for 5 cycles. **d** ICG and FA-IHA NPs fluorescence change over 30 days

Encouraged by the strong NIR optical absorption of FA-IHA NPs, the photothermal property of FA-IHA NPs was evaluated. Water, free ICG, and FA-IHA NPs (with equal ICG concentration) were irradiated with an 808 nm laser (1 W/cm^2^). The temperature of the FA-IHA NPs and free ICG increased by approximately 36 °C within 5 min of irradiation (Fig. 3a), while water gave a temperature increment less than 4 °C, demonstrating that the ICG-contained nanoparticles have significant photothermal effect and have the potential for cancer therapy. In addition, Additional file 1: Figure S1 shows the photothermal heating curves of FA-IHA NPs under 5 min of 808 nm laser irradiation with 0.5, 1, and 1.5 W/cm^2^, indicating that the optimum laser intensity is 1 W/cm^2^. The photostability tests on FA-IHA NPs and free ICG were performed. Free ICG showed a significant temperature decrease after five cycles compared to FA-IHA NPs (Fig. 3b). Figure 3c shows the absorption intensity change of free ICG and FA-IHA NPs before and after five cycles of NIR irradiation (808 nm, 1 W/cm^2^). The results suggested that the absorbance intensity at 808 nm of free ICG decreased after five cycles of NIR irradiation, while FA-IHA NPs maintained the pristine absorbance intensity. In addition, we compared the fluorescence stability of free ICG and FA-IHA NPs (Fig. 3d). After 30 days storage at 4 °C, the fluorescence intensity of FA-IHA NPs at 800 nm was 0.72 compared to its initial intensity of 1, while the fluorescence of free ICG dropped to 0.12 compared to its initial intensity, due to the aggregation induced-photobleaching [36]. These results indicated that the covalently conjugated ICG was more stable than the free ICG, likely due to the HSA and PEG self-assembly protecting ICG from internal environment-induced aggregation, such as heat or light. Thus, these results suggested that FA-IHA NPs had excellent photothermal effect and photothermal stability.

**Fig. 4:**
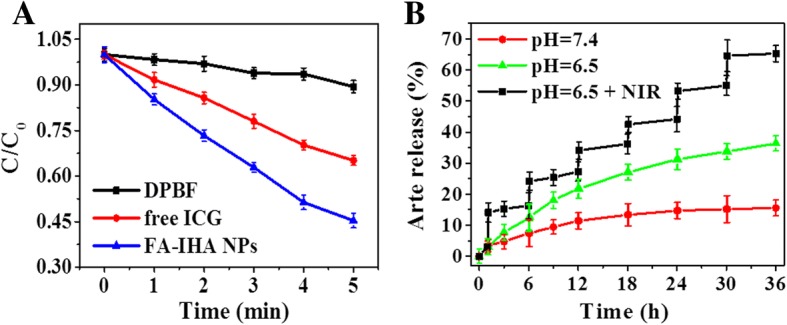
**a** Normalized DPBF absorbance in the presence of ICG, FA-IHA NPs, and blank sample under 808 nm laser irradiation (1 W/cm^2^). **b** Release kinetics of Arte from FA-IHA NPs under pH = 7.4 and pH = 6.5 with or without NIR laser irradiation, respectively

Next, a ROS-specific probe 1,3-diphenylisobenzofuran (DPBF) was used to detect ROS production by FA-IHA NPs after NIR irradiation. As shown in Fig. 4a, FA-IHA NPs produced a significant ROS amount (0.58 in the standard absorbance) within 5 min of NIR irradiation compared to free ICG (0.35), which might be attributed to FA-IHA NPs combination therapy.

Under NIR laser irradiation (808 nm, 1 W/cm^2^) and pH condition, the release performance was investigated (Fig. 4b). As the contrast, without NIR irradiation, FA-IHA NPs showed 11.61% and 34.2% Arte release under pH 7.4 and pH 6.5, respectively, while under NIR irradiation for six times, FA-IHA NPs showed a total of 68.4% Arte release at pH 6.5, suggesting that NIR irradiation and acid condition could both significantly trigger Arte release from FA-IHA NPs. The NIR irradiation and acid responsive drug release were likely due to the heat-induced expansion of HSA nanoparticles, and in addition, at acidic environment, the H^+^ could change the surface charge of HSA that alter the hydrophilic/hydrophobic balance of the nanoparticles [37, 38].

### Cell Uptake and Detection of Intracellular ROS

**Fig. 5:**
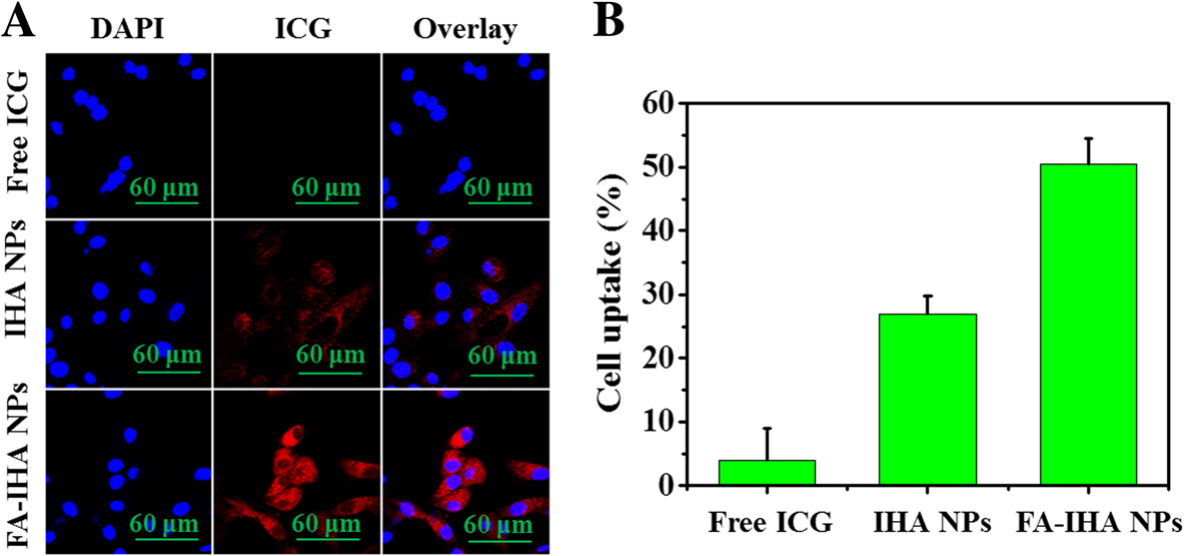
**a** Confocal fluorescent images of HepG2 cells after incubation with free ICG and IHA NPs, and FA-IHA NPs. Red and blue colors represent ICG fluorescence and DAPI-stained cell nuclei, respectively. **b** Flow cytometry measurement of ICG fluorescence intensities in HepG2 cells after incubation with free ICG and IHA NPs and FA-IHA NPs

Thanks to ICG fluorescence properties, FA-IHA NPs uptake was directly observed in HepG2 cells through a fluorescence microscope. As shown in Fig. 5a, after treatment of the cells with FA-IHA NPs, the cytoplasm showed stronger red ICG fluorescence than that observed in cells treated with free ICG and IHA NPs. Furthermore, FA-IHA NPs cell uptake ratio was quantitated by FCM as 52.3%, which was higher than that of IHA NPs (25.2%) and free ICG (3.9%) (Fig. 5b). The results demonstrated that the conjugated FA facilitated the nanoparticles to target the FA receptors on tumor cells and thus enhance FA-IHA NPs cell uptake [39, 40, 41].

**Fig. 6:**
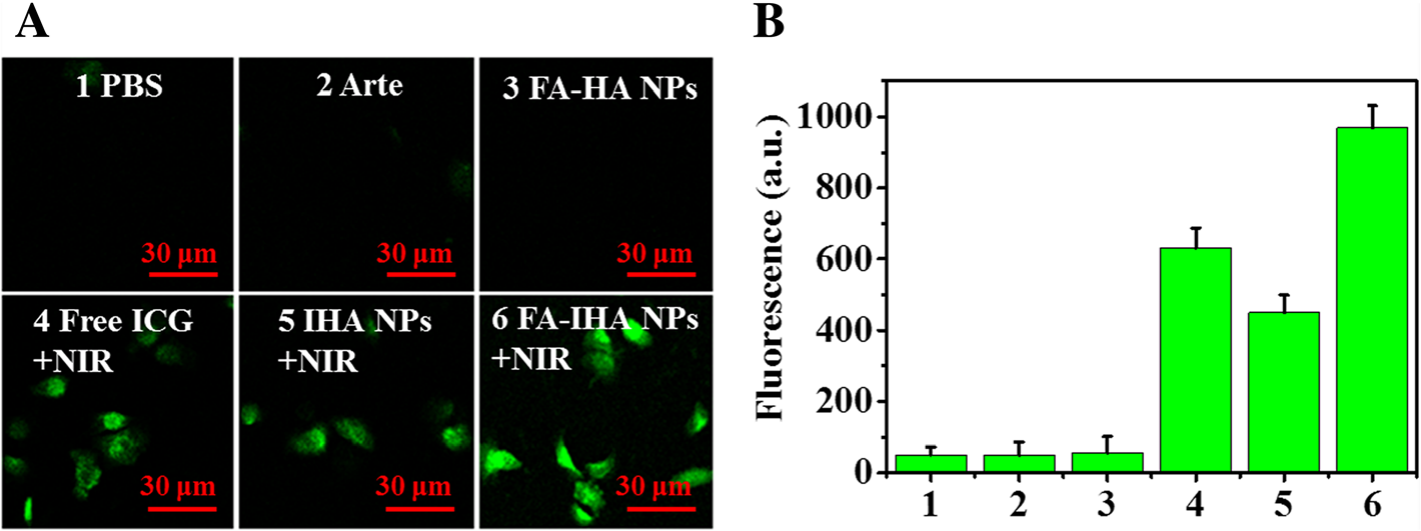
**a** Fluorescence images of ROS production in cancer cells treated with various drugs, and **b** corresponding fluorescent intensity: (1) PBS, (2) Arte, (3) FA-HA NPs, (4) free ICG + NIR, (5) IHA NPs + NIR, and (6) FA-IHA NPs + NIR

By using a fluorescence microscope, we observed the intrinsic photodynamic activity of Arte-, ICG-, and FA-IHA NPs-treated cells with or without NIR irradiation. A ROS probe 2, 7-dichlorodihydrofluorescein diacetate was used to visualize cellular ROS production. The results showed that FA-IHA NPs could induce a significantly enhanced ROS production compared with other samples after 5 min of NIR irradiation (Fig. 6a). The corresponding fluorescent values are shown in Fig. 6b.

### In Vitro Tumor Combinational Photo-Chemo therapy

**Fig. 7:**
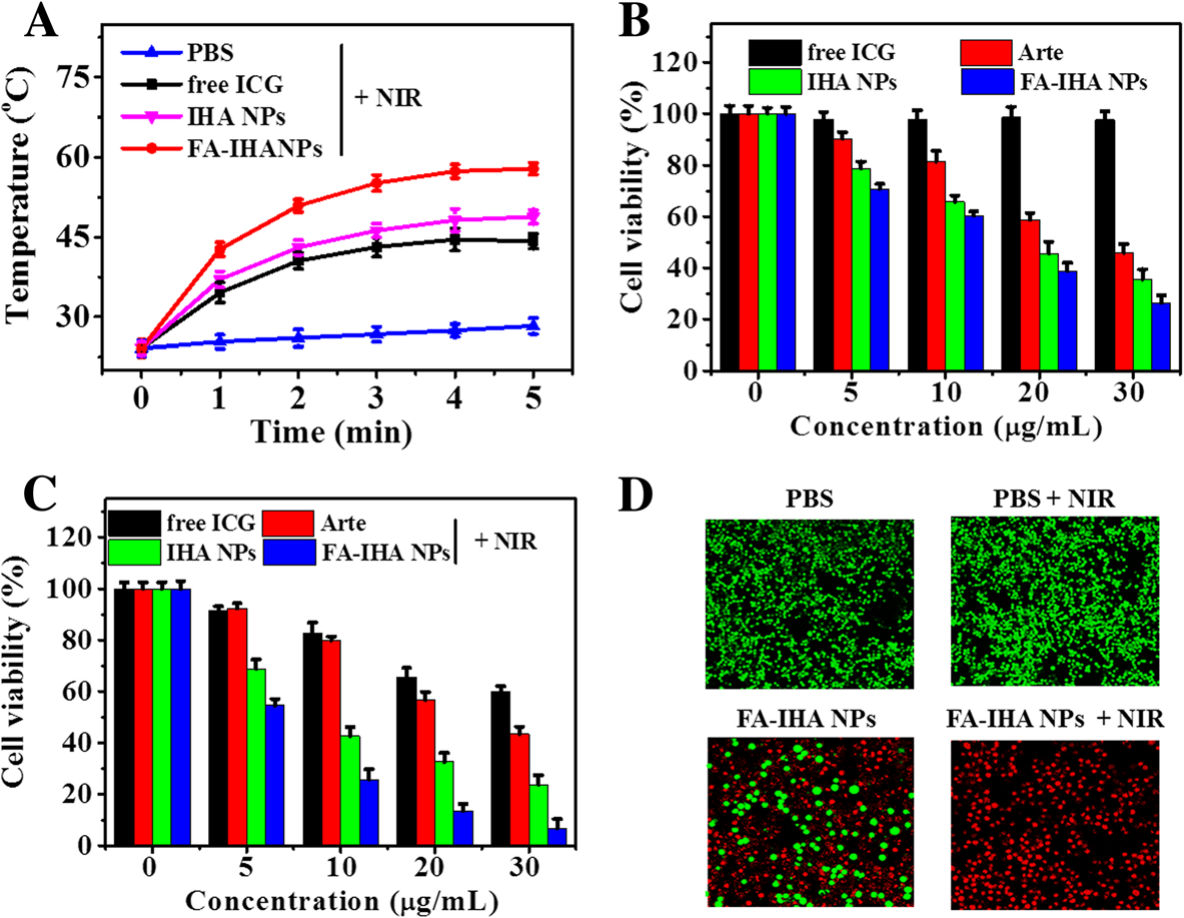
**a** Temperature change curves of PBS-, free ICG-, IHA NPs-, and FA-IHA NPs-treated cells in 96-well plates after 5 min of NIR irradiation. **b, c** Cell viability of cells treated with free ICG, Arte, IHA NPs, and FA-IHA NPs without or with 808 nm laser irradiation (5 min, 1 W/cm^2^), respectively. **d** Calcium AM/PI dual-staining images of cells after treatment with PBS (control), PBS + NIR, FA-IHA NPs, and FA-IHA NPs + NIR, respectively

Figure 7a shows the temperature change of the PBS-, free ICG-, IHA NPs-, and FA-IHA NPs-treated cells (with equal ICG concentration) after 5 min of NIR irradiation (1.0 W/cm^2^). The temperature of cells treated with FA-IHA NPs showed the highest increase (*ΔT* = 31 °C) compared with that of PBS-, free ICG-, and IHA NPs-treated cells. The viability of cells treated with Arte, IHA NPs, and FA-IHA NPs at different concentrations for 24 h without NIR irradiation decreased with increased concentration, while free ICG at these concentrations did not show any cytotoxicity (Fig. 7b). Meanwhile, the drug carrier FA-IH NPs (FA-IHA NPs without Arte) also showed no significant cytotoxicity (Additional file 1: Figure S2). In contrast, after NIR irradiation (1.0 W/cm^2^, 5 min), significant concentration-dependent cell death was observed in cells treated with free ICG, IHA NPs, and FA-IHA NPs (Fig. 7c). The effect was particularly significant in FA-IHA NPs-treated cells. The excellent anticancer effect might be attributed by the targeted combinational photo-chemo therapy, such as the chemotherapeutic effect of the released Arte and the PTT-PDT therapeutic effect of ICG. Furthermore, the cytotoxicity of FA-IHA NPs with or without NIR irradiation was investigated by calcein-AM/PI dual staining. Cells treated with FA-IHA NPs and irradiation were almost completely dead compared to other treated groups (Fig. 7d).

### In Vivo Fluorescence Imaging

**Fig. 8:**
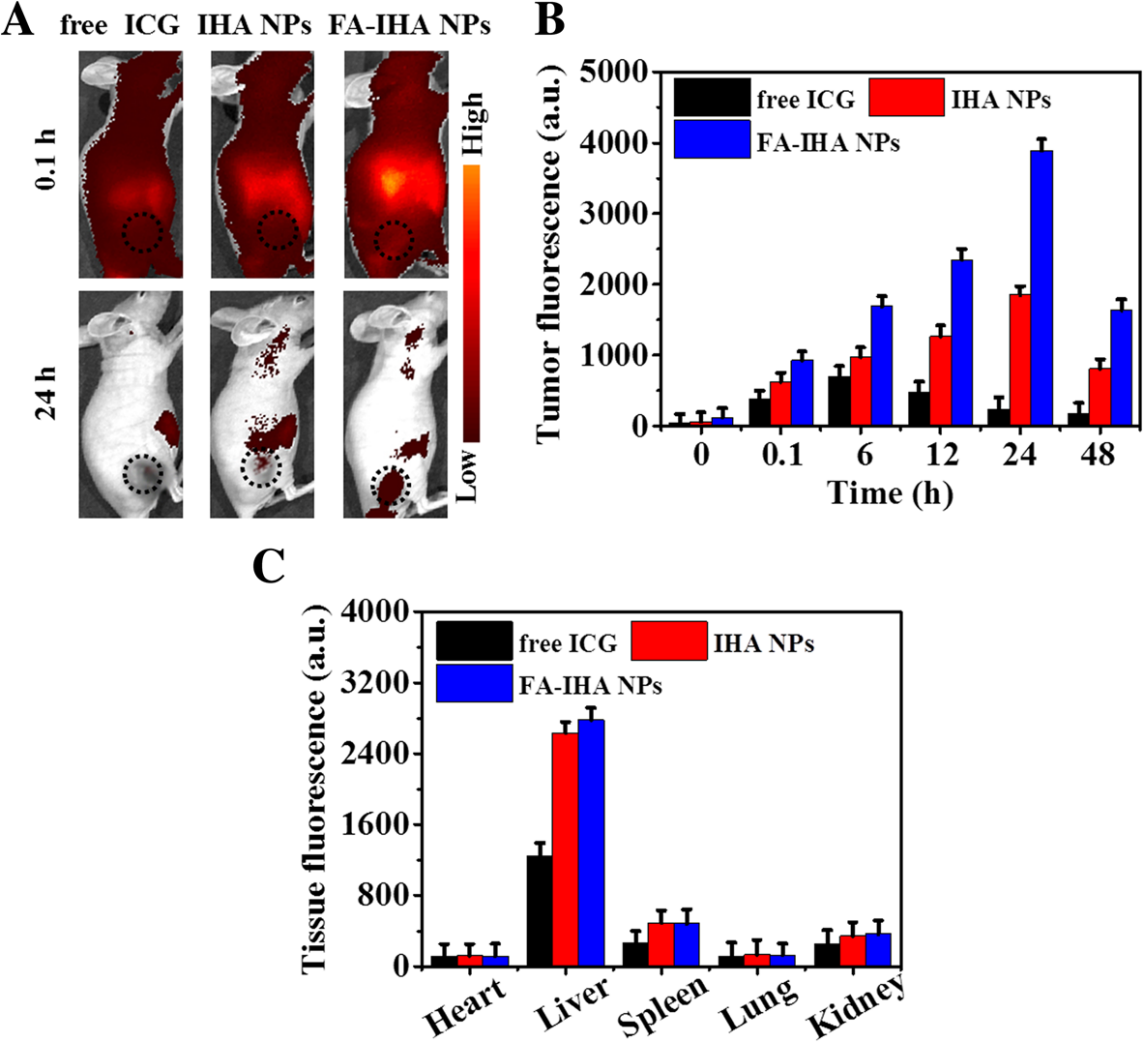
**a** Representative fluorescence images of tumor-bearing mice after tail vein injection with free ICG, IHA NPs, and FA-RIPNPs. The black dashed circles indicate the tumor region. **b** Quantitative in vivo analysis of the fluorescence signal in the tumor regions of mice treated with free ICG, IHA NPs, and FA-IHA NPs as a function of injection time. **c** The fluorescence signal of major organs including heart, liver, spleen, lungs, and kidney

As shown in Fig. 8a and b 0.1 h post-injection of free ICG, IHA NPs, and FA-IHA NPs, a strong fluorescence signal could be seen in the entire body of the tumor-bearing mice. Fluorescence signals increased in the tumor region with the increasing time, reaching the peak at 24 h post-injection. The tumor fluorescence signals in the FA-IHA NPs group were the highest compared to that of ICG and IHA NPs groups at all tested points (Fig. 8b), indicating that FA-IHA NPs could highly accumulate in the tumor region due to the FA-induced tumor-targeted effect. Additionally, the biodistribution in major tissues, including heart, liver, spleen, lungs, and kidneys, was conducted by an ex vivo fluorescence quantitative analysis 24 h post-injection. In all tested groups, strong fluorescence signals were detected in the liver tissue (Fig. 8c), indicating that the main metabolic conversion of these compounds follows a hepatic pathway. These results demonstrated that FA-IHA NPs could selectively accumulate in tumors in vivo, likely induced by the FA-targeted effect [37].

### In Vivo Tumor Combinational Photo-Chemo therapy

**Fig. 9:**
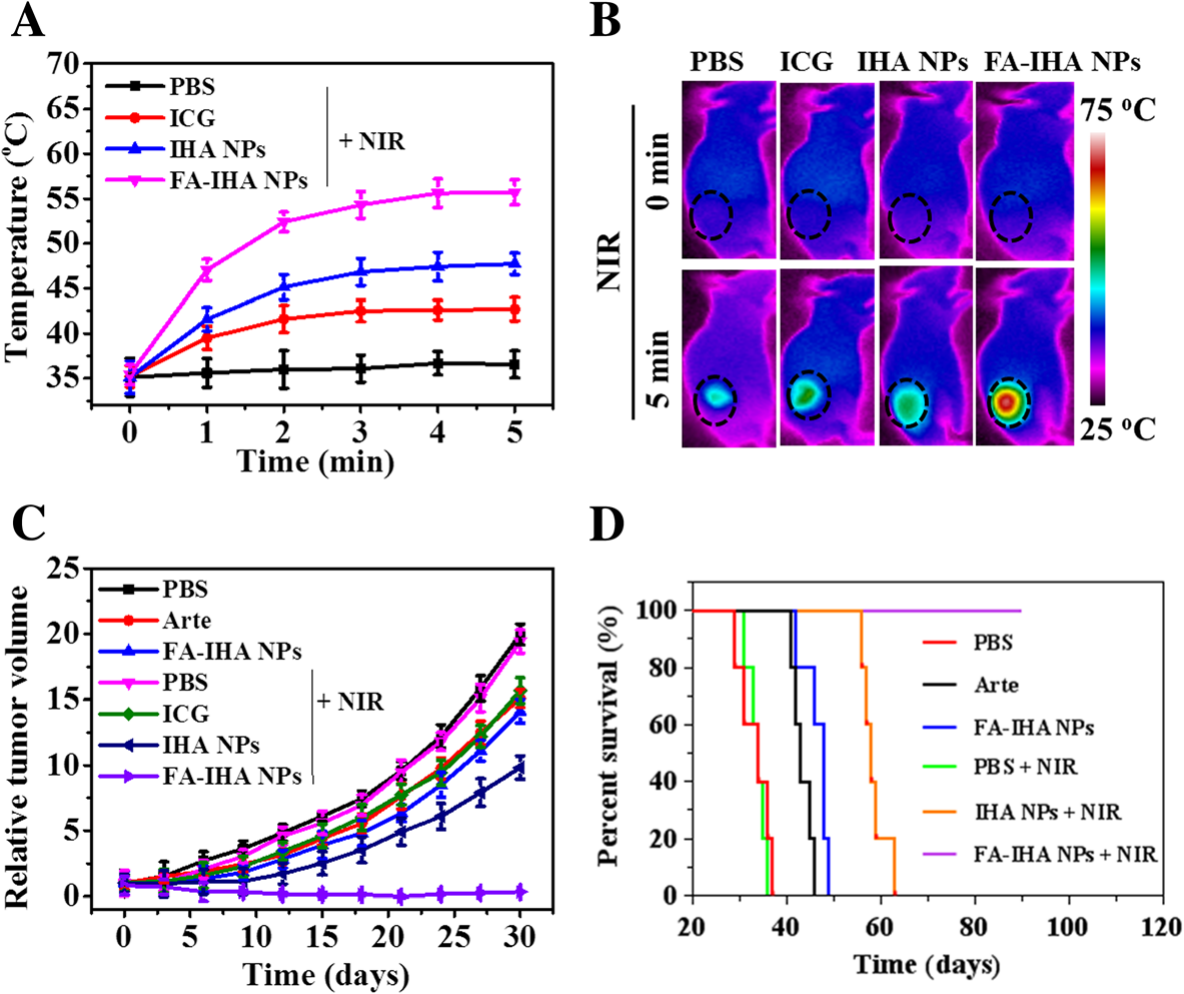
**a** Tumor region temperatures in tumor-bearing mice after tail vein injection of PBS, ICG, IHA NPs, and FA-RIPNPs at 24 h under 5 min of NIR irradiation (808 nm, 1 W/cm^2^). **b** Thermal images of tumor-bearing mice after tail vein injection of PBS, ICG, IHA NPs, and FA-RIPNPs at 24 h under 5 min of NIR irradiation (808 nm, 1 W/cm^2^). **c** HepG2 xenograft tumors growth profile after intravenous injection of PBS, Arte, ICG, IHA NPs, and FA-RIPNPs with or without 5 min of NIR irradiation (808 nm, 1 W/cm^2^). **d** Survival rate of tumor-bearing mice after tail vein injection with PBS, free Arte, ICG, IHA NPs, and FA-RIPNPs with or without 5 min of NIR irradiation (808 nm, 1 W/cm^2^)

As shown in Fig. 9a and b, the tumor temperature in the tumor-bearing mice after treatment with PBS, free ICG, IHA NPs, and FA-IHA NPs at 24 h post-injection under 5 min of NIR irradiation (1 W/cm^2^) was recorded by a thermal imager. An approximate 22.1 °C increase of the tumor region was detected in FA-IHA NPs-treated group, which was the highest than that of other groups. After two cycles of NIR irradiation (day 0 and day 2), FA-IHA NPs + NIR group exhibited a significant tumor growth suppression without a relapse (Fig. 9c), while the groups treated with PBS, Arte, FA-IHA NPs, PBS + NIR, ICG + NIR, and IHA NPs + NIR exhibited no clear indication of tumor suppression. Furthermore, after 90 days, the mice in FA-IHA NPs + NIR group showed 100% survival rate (Fig. 9d). These results indicate that FA-IHA NPs with NIR irradiation had an excellent in vivo tumor therapeutic efficacy, likely due to active targeted and combination photo-chemo therapy.

**Fig. 10:**
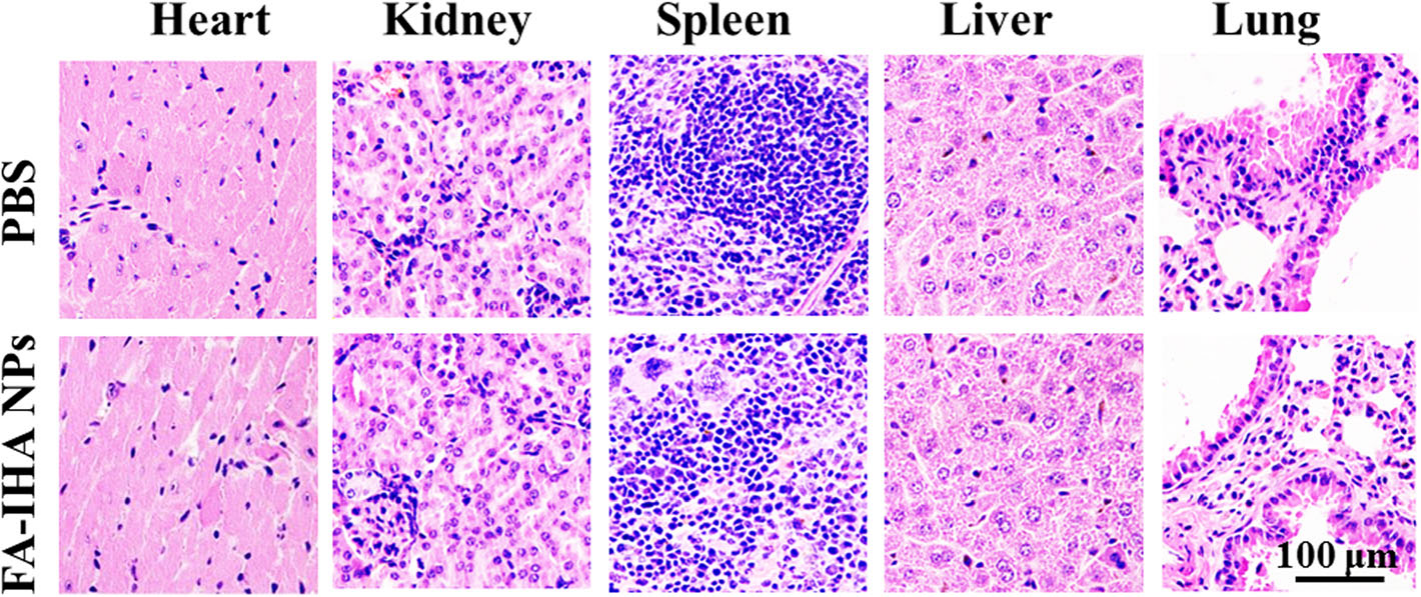
H&E-stained tissue sections of major organs, including heart, liver, spleen, lungs, and kidney from mice treated with PBS and FA-IHA NPs

Finally, hematoxylin and eosin (H&E) staining was used to evaluate FA-IHA NPs toxicity. The section images showed no significant histological lesions compared to PBS-treated group (Fig. 10), indicating FA-IHA NPs had neglectable toxicity, which was likely due to the safety of the ingredients of the FA-IHA NPs, thus being beneficial for their future use in clinical practice.

## Conclusions

In conclusion, a multifunctional theranostic agent was prepared, covalently conjugating FA and ICG, and encapsulating Arte for imaging-guided tumor-targeted combinational photo-chemo therapy in vitro and in vivo. The prepared FA-IHA NPs showed excellent colloidal- and heat-stabilities and fluorescence property. Under NIR irradiation, FA-IHA NPs showed great photothermal effect which could trigger Arte release and produce much more ROS after NIR irradiation than free ICG that exhibited photodynamic performance. The conjugated FA facilitated a highly efficient cellular uptake and tumor accumulation in vitro and in vivo. Moreover, the highly effective anticancer efficacy of FA-IHA NPs combined active targeting thermal drug chemotherapy, such as PTT-PDT therapy, which was demonstrated in vitro and in vivo. Overall, the results obtained indicated that FA-IHA NPs might be a promising tumor-targeted system feasible for future nanomedicine applications.

## Additional File

Additional file 1: **Figure S1.** Photothermal heating curves of FA-IHA NPs under 5 min 808 nm laser irradiation with 0.5, 1 and 1.5 W/cm^2^. **Figure S2.** Cell viabilities of HepG 2 cells after incubation with different concentration of the drug carrier FA-IH NPs (FA-IHA NPs without Arte). (DOCX 30 kb)

## Abbreviations

Arte: Artesunate
FA: Folic acid
HSA: Human serum albumin
ICG: Indocyanine green
NIR: Near infrared
PDT: Photodynamic therapy
PEG: Polyethylene glycol
PTT: Photothermal therapy
