# Supplementary material for: Artesunate-Loaded and Near-Infrared Dye-Conjugated Albumin Nanoparticles as High-Efficiency Tumor-Targeted Photo-Chemo Theranostic Agent
Source: Nanoscale Res Lett. 2018 Oct 11;13:319. doi: 10.1186/s11671-018-2700-5 (PMC6181830; doi:10.1186/s11671-018-2700-5)
Supplement: Supplementary file 1 — Figure S1. Photothermal heating curves of FA-IHA NPs under 5 min 808 nm laser irradiation with 0.5, 1 and 1.5 W/cm2. Figure S2. Cell viabilities of HepG 2 cells after incubation with different concentration of the drug carrier FA-IH NPs (FA-IHA NPs without Arte). (DOCX 30 kb) [file 11671_2018_2700_MOESM1_ESM.docx]

**Supplementary Material**

**Artesunate loaded and near infrared dye conjugated albumin nanoparticles as high-efficiency tumor targeted photo-chemo theranostic agent**

**Hainan Yang^1†^, Zaijia Liu^2†^, Xufeng Li^1^, Zhenfeng Zhang^1^, Deji Chen^1*^, Hui Lian^1*^**

^1^ Department of Radiology, the Second Affiliated Hospital of Guangzhou Medical University, Guangzhou 510260, China

^2^ Department of Medical Image, Ezhou Central Hospital, Ezhou 436000, China

^†^ These authors contribute equally to this work

^*^ Correspondence:

Deji Chen, E-mail: dengwt_clin@163.com; Tel: +86-020-34153532

Hui Lian, E-mail: lianhui_gzmu_edu@hotmail.com; Tel: +86-13725307026


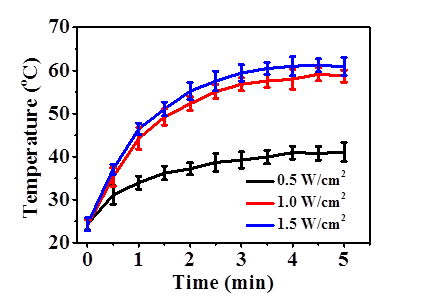


**Figure S1.** Photothermal heating curves of FA-IHA NPs under 5 min 808 nm laser irradiation with 0.5, 1 and 1.5 W/cm^2^.


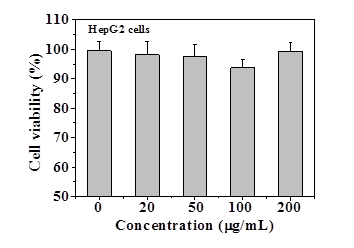


**Figure S2.** Cell viabilities of HepG 2 cells after incubation with different concentration of the drug carrier FA-IH NPs (FA-IHA NPs without Arte).
